# HAMAMATSU-ICG study: Protocol for a phase III, multicentre, single-arm study to assess the usefulness of indocyanine green fluorescent lymphography in assessing secondary lymphoedema

**DOI:** 10.1016/j.conctc.2020.100595

**Published:** 2020-06-16

**Authors:** Shinsuke Akita, Naoki Unno, Jiro Maegawa, Yoshihiro Kimata, Hidekazu Fukamizu, Yuichiro Yabuki, Akira Shinaoka, Masaki Sano, Yohei Kawasaki, Tadami Fujiwara, Hideki Hanaoka, Nobuyuki Mitsukawa

**Affiliations:** a Department of Plastic, Reconstructive, and Aesthetic Surgery, Chiba University Graduate School of Medicine, Chiba, Japan; b Department of Vascular Surgery, Hamamatsu Medical Center, Hamamatsu, Japan; c Second Department of Surgery, Hamamatsu University School of Medicine, Hamamatsu, Japan; d Department of Plastic and Reconstructive Surgery, Yokohama City University, Graduate School of Medicine, Yokohama, Japan; e Department of Plastic and Reconstructive Surgery, Okayama University Graduate School of Medicine, Dentistry and Pharmaceutical Science, Okayama, Japan; f Department of Plastic and Reconstructive Surgery, Hamamatsu University School of Medicine, Hamamatsu, Japan; g Clinical Research Center, Chiba University Hospital, Chiba, Japan

**Keywords:** Indocyanine green fluorescent lymphography, Secondary lymphoedema, Lymphaticovenular anastomosis

## Abstract

**Introduction:**

Secondary lymphoedema of the extremities is an important quality-of-life issue for patients who were treated for their malignancies. Indocyanine green (ICG) fluorescent lymphography may be helpful for assessing lymphoedema and for planning lymphaticovenular anastomosis (LVA). The objective of the present clinical trial is to confirm whether or not ICG fluorescent lymphography using the near-infrared monitoring camera is useful for assessing the indication for LVA, for the identification of the lymphatic vessels before the conduct of LVA, and for the confirmation of the patency of the anastomosis site during surgery.

**Methods and analysis:**

This trial is a phase III, multicentre, single-arm, open-label clinical trial to assess the efficacy and safety of ICG fluorescent lymphography when assessing and treating lymphoedema of patients with secondary lymphoedema who are under consideration for LVA. The primary endpoint is the identification rate of the lymphatic vessels at the incision site based on ICG fluorescent lymphograms obtained before surgery. The secondary endpoints are 1) the sensitivity and specificity of dermal back flow determined by ICG fluorescent lymphography as compared with ^99m^Tc lymphoscintigraphy—one of the standard diagnostic methods and 2) the usefulness of ICG fluorescent lymphography when confirming the patency of the anastomosis site after LVA.

**Ethics and dissemination:**

The protocol for the study was approved by the Institutional Review Board of each institution. The trial was filed for and registered at the Pharmaceuticals and Medical Devices Agency in Japan. The trial is currently on-going and is scheduled to end in June 2020.

**Trial registration number:**

jRCT2031190064; Pre-results.

## Highlights

• This study will examine the efficacy of a highly safe drug, indocyanine green for evaluating lymphatic anatomy.
• This is the first, multicentre, prospective, systematised clinical trial of Indocyanine green lymphography.
• The present clinical trial will not allow the assessment of the therapeutic efficacy of lymphaticovenular anastomosis.

## 1 Introduction

Secondary lymphoedema of the extremities is an important quality-of-life issue for patients who were treated for their malignancies [1,2]. Lymphoedema is generally difficult to treat, and conservative treatment is the main treatment [3,4]. ^99m^Tc lymphoscintigraphy has been used for many years as one of the standard diagnostic methods of lymphoedema and is currently covered by the national health insurance of Japan [[5], [6], [7]]. However, lymphoscintigraphy has the following disadvantages: high costs, large-sized equipment for examination, low resolution, and radiation exposure.

Since 2007, fluorescent lymphography with indocyanine green (ICG) for assessing lymphoedema has been introduced [[8], [9], [10], [11], [12], [13], [14], [15], [16], [17], [18], [19], [20], [21], [22], [23], [24], [25]]. ICG fluorescent lymphography has been reported as a potentially useful assessment method to depict the lymphatic vessels in real time and to assess their function at low cost. ICG fluorescent lymphography allows the monitoring of time-course changes in the morphology and function of the lymphatic vessels and can be conducted anywhere including the examination and operation rooms [[8], [9], [10], [11], [12]]. A retrospective study showed a significant correlation in lymphoedema severity between ICG fluorescent lymphography and lymphoscintigraphy [10]. The usefulness of lymphaticovenular anastomosis (LVA) is well recognized among lymphatic microsurgeons, and ICG fluorescent lymphography has evolved as a reliable assessment method of extremity lymphoedema. In recent years, the usefulness of LVA that improves lymphatic flow has been reported [[26], [27], [28]].

ICG fluorescent lymphography using the near-infrared monitoring camera utilizes the following properties: 1) ICG is excited by irradiating the near-infrared ray of 760 nm in wavelength, and fluorescence of 830 nm in wavelength is thus generated; and 2) ICG, which was injected subcutaneously, is mostly bound to albumin and is then incorporated and delivered into the lymphatic vessels [8]. The use of the near-infrared monitoring camera permits the demonstration of the current direction of ICG in the lymphatic vessels and of the regurgitation of the lymphatic valves, thus allowing the depiction of dermal back flow—a finding characteristic of lymphoedema. Furthermore, the camera quite excels in allowing the real-time confirmation of lymph dynamic. Some studies reported a significant correlation between pathological stages determined by ^99m^Tc lymphoscintigraphy and ICG fluorescent lymphography; however, the ICG findings appropriate for LVA have not been standardized [[10], [11], [12]].

ICG fluorescent lymphography is expected to help the appropriate selection of patients with lymphoedema who are indicated for LVA, to serve for the identification of the lymphatic vessels before and during surgery and for the confirmation of the patency of the anastomosis site, and to greatly contribute to an improvement in the therapeutic outcomes of patients with lymphoedema; however, all of them need to be investigated in a prospective clinical study.

The objective of the present clinical trial is to confirm whether or not ICG fluorescent lymphography using the near-infrared monitoring camera is useful for specifying patients to whom LVA is indicated, for lymphatic vessel identification before the conduct of LVA, and for the confirmation of anastomosis site patency during surgery.

## 2 Methods

### 2.1 Trial design

The present clinical trial is a multicentre, collaborative, confirmatory, single-arm, open-label study. All patients with secondary lymphoedema in the lower or upper extremities, who are under consideration for LVA at the participating institutions, will receive written and oral information about the HArmonised Medical Assessment of lyMphoedemA based on The diagnoStic Usefulness of fluorescent lymphography with IndoCyanine Green (HAMAMATSU-ICG) study.

**Fig. 1:**
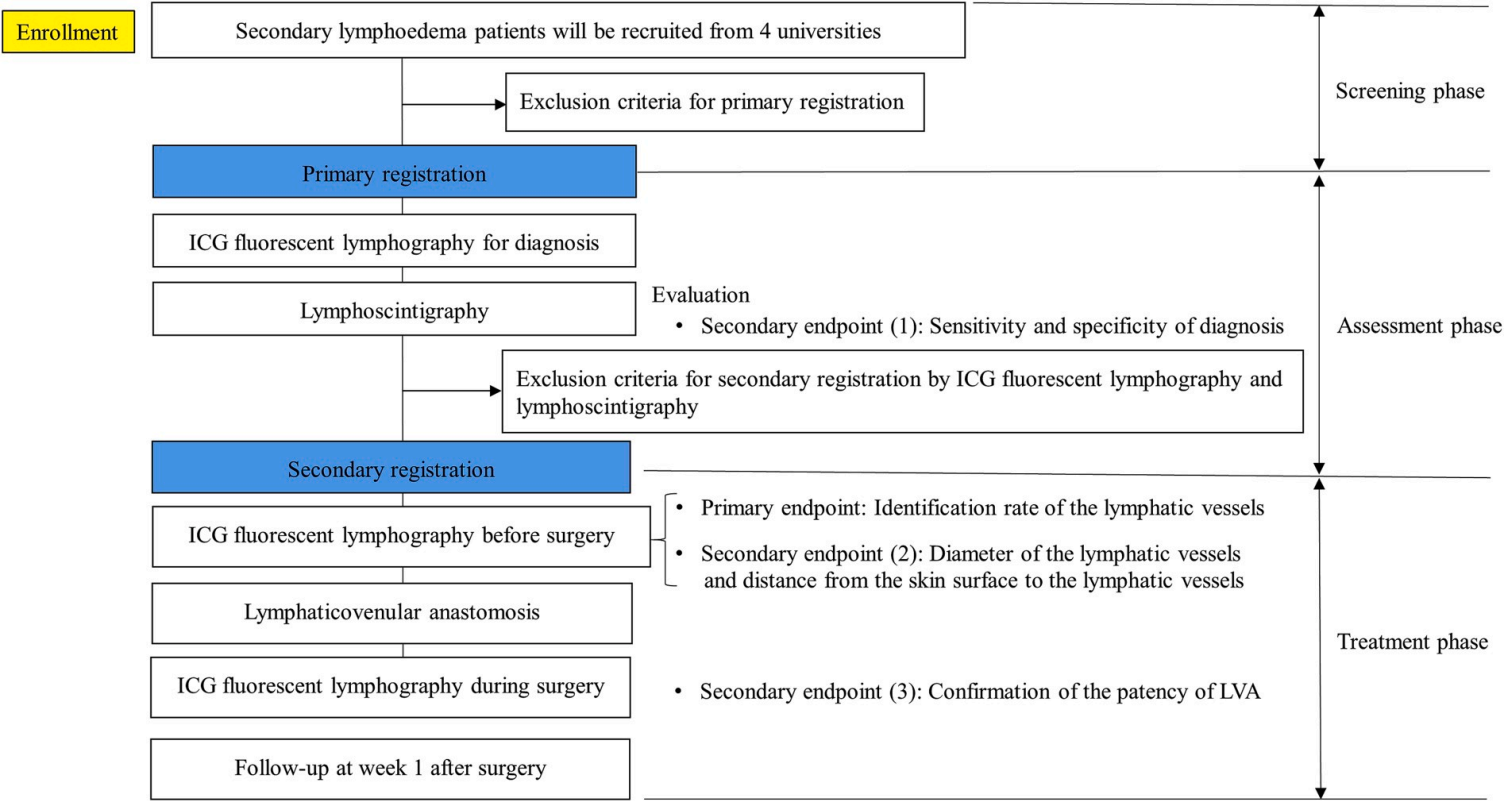
Diagram of the study outline. ICG, indocyanine green; LVA, lymphaticovenular anastomosis. (For interpretation of the references to colour in this figure legend, the reader is referred to the Web version of this article.)

In this study, the primary endpoint can be verified only in patients with radiographic evidence of lymphostasis and the presence of the anastomosable lymphatic vessels, who are candidates for LVA. Therefore, patients who meet the inclusion criteria and do not fall under any of the exclusion criteria for this study should receive investigational drugs after registration to assess the presence of lymphedema and anastomosable lymphatics. Two types of registration are established in this study: the primary registration—the registration of patients to be conducted at the onset of the assessment phase; and the secondary registration—the registration of patients to be conducted at the onset of the treatment phase. After the primary registration, patients should be evaluated in the assessment phase, and those who meet the inclusion criteria and do not fall under any of the exclusion criteria for the secondary registration should be evaluated in the treatment phase. Patients who meet the eligibility criteria for primary registration should undergo ICG fluorescent lymphography and ^99m^Tc lymphoscintigraphy. LVA should not be conducted for ineligible patients among them, and the present study should be discontinued for the former. Patients who meet the eligibility criteria for secondary registration will undergo preoperative and intraoperative ICG fluorescent lymphography. The trial outline is schematised in Fig. 1.

### 2.2 Eligibility criteria

A patient, who meets all of the following conditions, should be subject to the primary registration: 1) who was diagnosed with secondary lymphoedema of the extremities based on clinical symptoms; 2) who was diagnosed with stage I to II lymphoedema from their clinical symptoms as defined by the International Society of Lymphology, which was categorized from stage 0-III; 3) who was 20 years of age or older at the time of informed consent acquisition; and 4) who gave his/her free will-based written informed consent after receiving a sufficient explanation prior to enrollment in the present clinical trial and based on a sufficient understating about the present clinical trial.

A patient, who falls under any of the following conditions, should not be subject to the primary registration: 1) who has primary lymphoedema; 2) who has allergy against iodine; 3) who has shown shock caused by ICG administration in the past; 4) who has shown symptoms of allergy caused by ^99m^Tc administration in the past; 5) who has allergy against xylocaine; 6) who is lean (body mass index [BMI]: <18 kg/m^2^) or obese (BMI: > 30 kg/m^2^); 7) who is a woman being pregnant, being breast-feeding, or willing to become pregnant during the study period; 8) who was otherwise considered inappropriate for enrollment in the clinical trial by the investigator; or 9) who has lymphoedema in both the upper and lower extremities, thus not allowing limitation to either the upper or lower extremities.

A patient, who meets all of the following conditions, should be subject to the secondary registration: 1) in whom the site of linear pattern was confirmed by ICG fluorescent lymphography after the primary registration; and 2) who was diagnosed with lymphoedema by ^99m^Tc lymphoscintigraphy after the primary registration.

A patient, whose ICG fluorescent lymphograms fall under any of the following conditions, should not be subject to the secondary registration: (1) in whom the linear pattern cannot be confirmed because of presenting only the “area of dermal back flow pattern that is indicative of the reversed current of the collecting lymphatic vessels into the skin” and the “area where a fluorescent lymphogram is not found at all,” which led to the judgment that the lymphatic vessels are difficult to identify; (2) who was otherwise judged to be inappropriate as a subject to the present trial; (3) who has had symptoms of allergy caused by the subcutaneous injection of ICG; or (4) who was diagnosed to have oedema due to causes other than lymphoedema because of presenting normal lymphoscintigraphic findings.

### 2.3 Recruitment

This trial was filed for and registered at the Pharmaceuticals and Medical Devices Agency (PMDA) in June 2019. Recruitment started in July 2019 and was completed in March 2020 because 55 patients were registered, which exceeded the target number of 50 patients with lymphoedema for the secondary registration. This study is being conducted at 4 university hospitals in Japan.

### 2.4 Sample size calculation

None of previous articles refer to the identification rates of the lymphatic vessels in patients with lymphoedema when conducting and not conducting ICG fluorescent lymphography. A multicentre clinical trial of ICG on sentinel lymph node biopsy in patients with breast cancer was designed to indicate its efficacy at a lymph vessel identification rate of 90%. Consequently, the lymph vessel identification rate was 99.6% [26].

ICG should be considered useful when the positivity rate is 90%. The target number of patients for the present clinical trial was calculated to be 118, when assuming a threshold of 80% and an expected value of 90% (90% power, 5% two-sided significance level).

Usually, LVA is conducted at 5 or more sites of an extremity. To avoid verifying the accuracy of lymphatic vessel identification several times for the same lymphatic vessels in a certain area, the extremity is divided into 4 areas and the number of examinations in each area is limited. Verification is expected to occur in 3 or more sites on average, and any upper limit is not established for the points to verify the accuracy of lymph node identification as long as the protocol is followed. The target number of patients with lymphoedema for the secondary registration, which is good enough to examine 150 sites, should be set at 50.

### 2.5 Interventions

ICG fluorescent lymphography should be conducted to achieve the following objectives according to the schedule for 3 active study visits:

#### 2.5.1 ICG fluorescent lymphography to assess lymphoedema in the ICG administration phase [assessment phase] after the primary registration

**Fig. 2:**
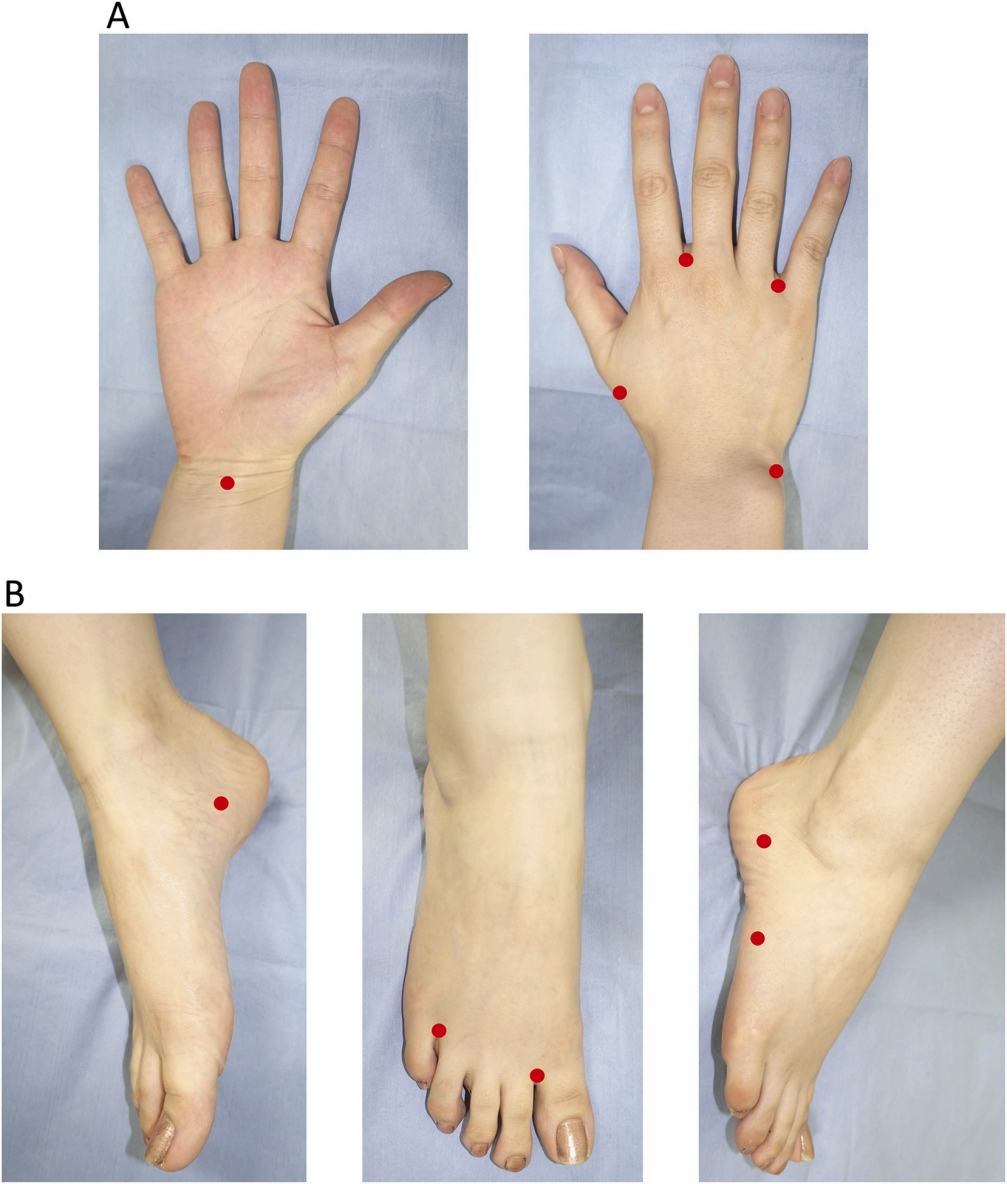
Five sites for ICG injection: (A) hand and (B) foot ICG, indocyanine green. (For interpretation of the references to colour in this figure legend, the reader is referred to the Web version of this article.)
2) With the patient on bed who is in the supine position and at rest and under indoor illumination that is reduced immediately after subcutaneous injection, the near-infrared monitoring camera (Pde-neo®, Hamamatsu Photonics K·K., Japan) will be used to monitor and record the imaging findings on the extremity surface. Regarding the lower extremities as well, recording should be conducted with the patient in the prone position. ICG, which is retained in the hypodermis, should be drained manually to quickly confirm the course of the collecting lymphatic vessels. ICG fluorescent lymphograms can be categorized to the following regions: A) the region of linear pattern where the pre-collecting lymphatic vessels or collecting lymphatic vessels are depicted in a linear pattern; B) the region of dermal back flow pattern where the reversed current of lymph of the collecting lymphatic vessels into the skin is obvious; and C) the region where any fluorescent image cannot be seen at all (Fig. 3). The image should be obtained immediately after injection. In patients in whom findings in the entire extremities cannot be obtained immediately after injection due to the slow movement of ICG, imaging should be conducted again at a later time without reinjection, and the time from injection to reimaging is recorded. However, the check of the findings should be completed not later than 3 h after injection.

1) Local anaesthesia with 0.5% xylocaine should be conducted beforehand at the planned site for ICG subcutaneous injection. The 2.5 mg/mL ICG solution (Diagnogreen®, 25 mg: Daiichi Sankyo Co., Ltd, Japan) should be injected subcutaneously to the right and left, upper or lower extremities. The target extremities should include 5 sites per extremity (upper extremities: the second interdigit, the fourth interdigit, the distal three-fourths point on the straight line that connects the distal end of the radial styloid process and the head of the first metacarpal bone, the distal end of the ulnar styloid process, and the distal forearm at the level of the tendon for long palmar muscle; and lower extremities: the first interdigit, the fourth interdigit, below the medial malleolus, below the lateral malleolus, and the midpoint on the straight line that connects the head of the fifth metatarsal bone and the lateral malleolus (Fig. 2) [27]. The 2.5 mg/mL ICG solution, 0.2 mL per site, should be injected subcutaneously.

**Fig. 3:**
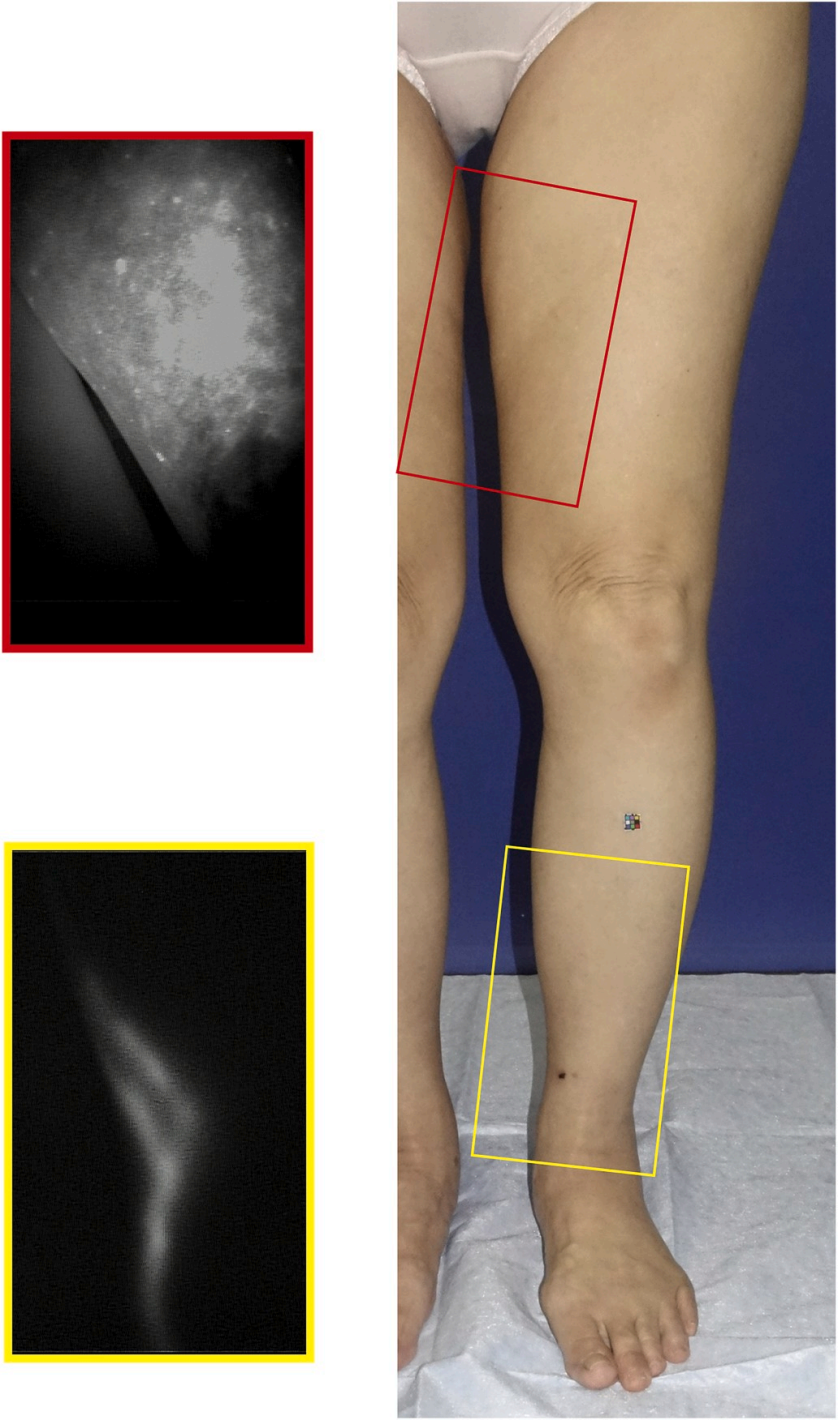
ICG fluorescent lymphograms can be categorized to the region of linear pattern, the region of dermal back flow pattern, and the region where any fluorescent image cannot be seen at all. (A): Linear pattern, (B): Dermal back flow pattern.ICG, indocyanine green. (For interpretation of the references to colour in this figure legend, the reader is referred to the Web version of this article.)
3) ^99m^Tc lymphoscintigraphy using the injection solution of a radiopharmaceutical agent, standard human serum albumin diethylenetriamine pentaacetic acid technetium (^99m^Tc) (Poolscinti® Injectable), should be conducted in all patients who were included in the primary registration within 8 weeks after ICG fluorescent lymphography in order to determine the sensitivity and specificity of lymphoedema diagnosis by ICG fluorescent lymphography when considering ^99m^Tc lymphoscintigraphy as one of the standard diagnostic methods. ^99m^Tc lymphoscintigraphy should be conducted at 120 min after radioisotope administration.

#### 2.5.2 Fluorescent ICG lymphography before surgery to identify the lymphatic vessels in the investigational drug administration phase [treatment phase] after the secondary registration

**Fig. 4:**
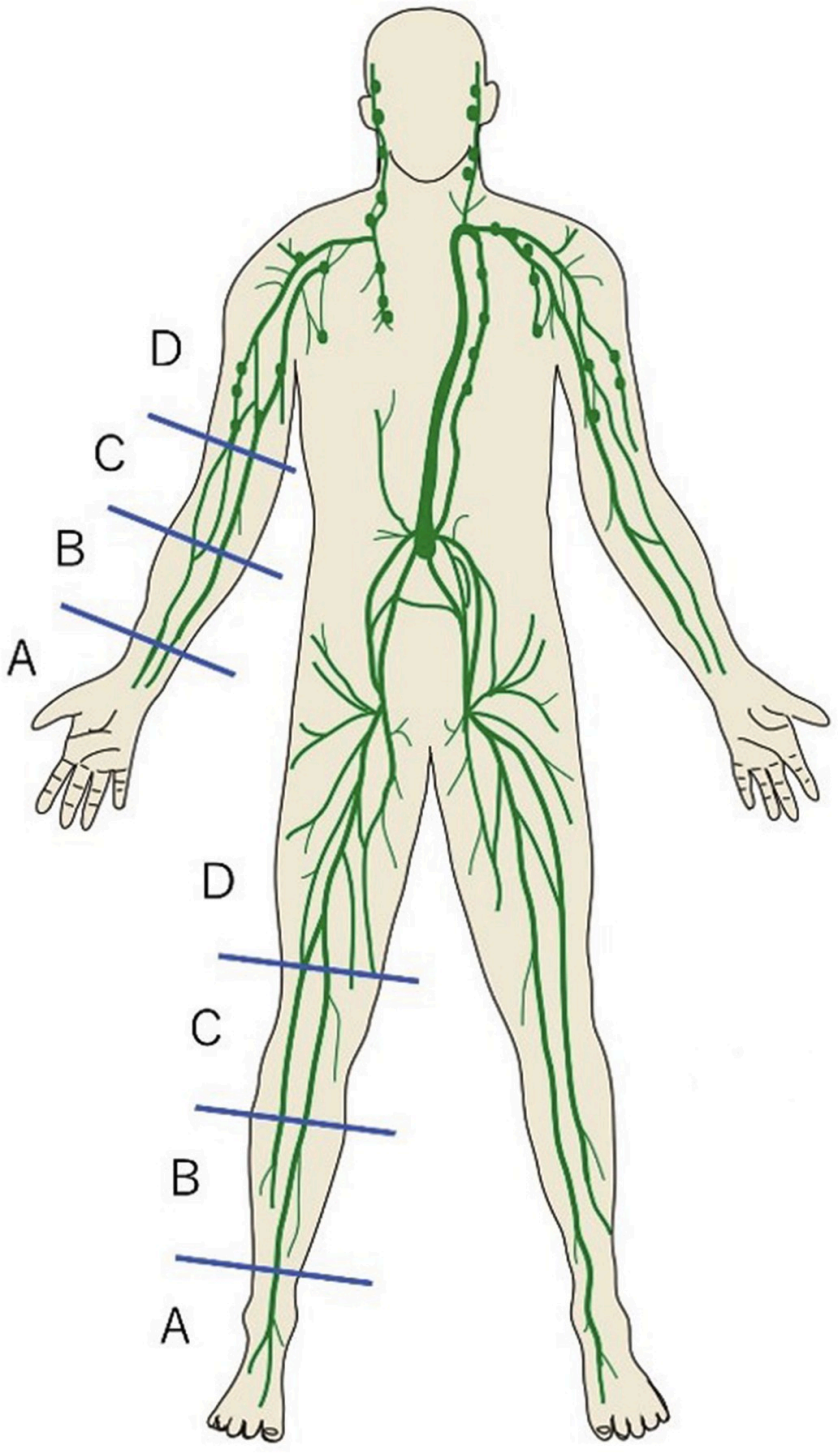
Both the upper and lower extremities were divided into the following 4 areas—lower extremities: 3 borders established at 6 cm proximal from the centre of the medial malleolus, 4 cm distal from the popliteal fossa, and 4 cm proximal from the popliteal fossa; and upper extremities—3 borders established at 6 cm proximal from the ulnar styloid process, 4 cm distal from the cubital fossa, and 4 cm proximal from the cubital fossa.

**Fig. 5:**
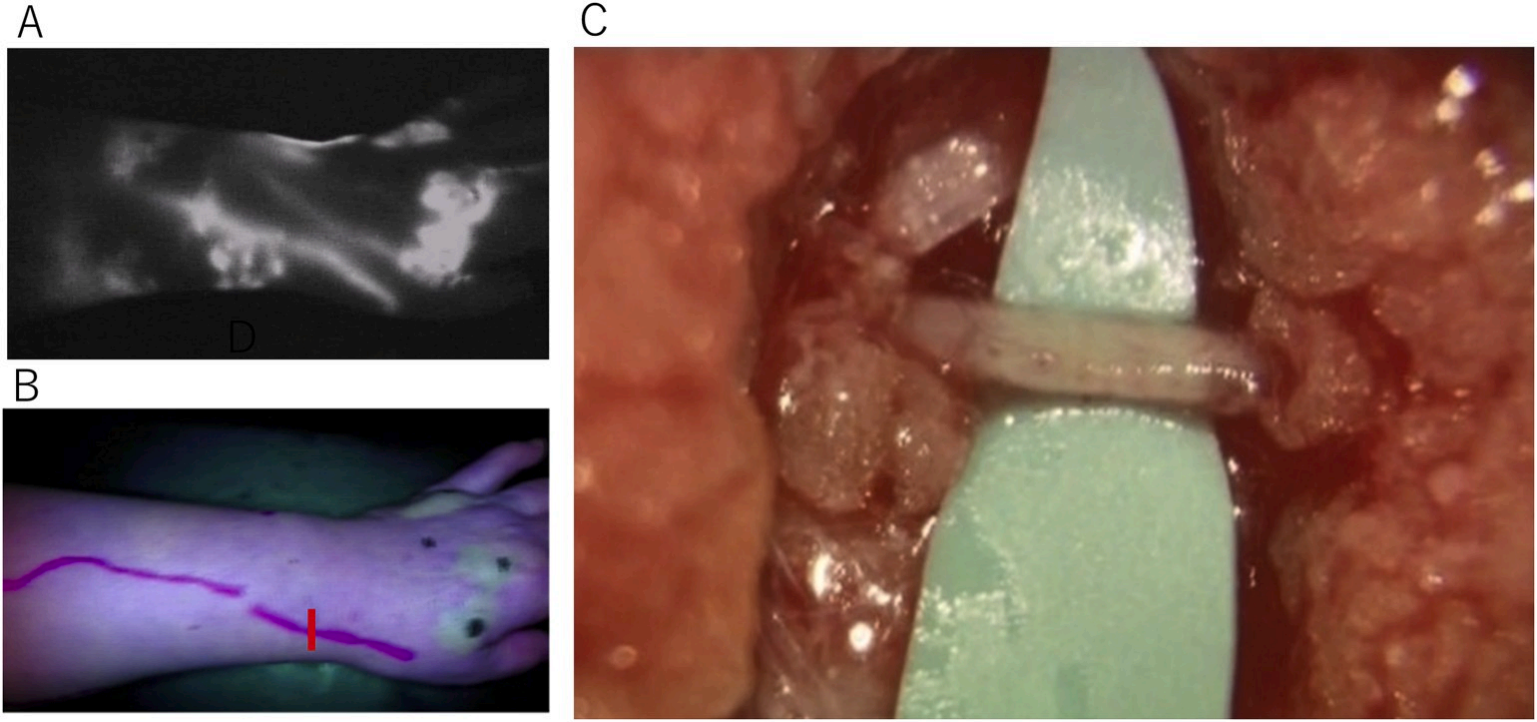
(A): ICG fluorescent lymphogram of the forearm and (B): an image of the same region under visible light. The skin incision site was determined preoperatively on the linear pattern of an ICG fluorescent lymphogram. (C): A photograph under visible light should be used to confirm whether the anastomosable collecting lymphatic vessels are successfully identified in reality on the line of skin incision.

1) Local anaesthesia with 0.5% xylocaine should be conducted beforehand at the planned sites for ICG subcutaneous injection. ICG should be injected subcutaneously to the extremity where LVA is intended. The target extremities should include 5 sites per extremity (Fig. 2). The 2.5 mg/mL ICG solution, 0.2 mL per site, should be injected subcutaneously.
2) With the patient on bed who is in the supine position after subcutaneous injection, a red pen should be used to mark the skin on the confirmed fluorescent lymphograms of linear pattern.
3) The incision site for LVA should be determined at the site where the linear pattern was found. The depicting ability of ICG is estimated to differ depending on the thickness of the skin and subcutaneous adipose tissue. Therefore, 4 areas should be established in distal-to-proximal order in both the upper and lower extremities (Fig. 4). The maximal number of skin incision sites for lymphatic vessel dissection should be 2 to avoid the repeated identification of the same lymphatic vessels in the same region. Furthermore, the skin incision site for research in the same area should be one for the continuing linear pattern. This arrangement is to avoid the mutual influences among multiple sites of anastomosis and to ensure their independency. The length of skin incision should be determined in response to the thickness of subcutaneous fat, ranging from 0.5 cm to 2.0 cm. Surgery should be conducted under general or local anaesthesia. The camera for visible light should be used to photograph and record the findings as to whether the anastomosable collecting lymphatic vessels are successfully identified in reality on the line of skin incision that was set before surgery (Fig. 5).

#### 2.5.3 ICG fluorescent lymphography during surgery to check the patency of the anastomosis site after LVA in the investigational drug administration phase [treatment phase]

LVA should be conducted in the case that the veins appropriate for LVA were confirmed in the periphery of the identified lymphatic vessels. After anastomosis, the surgeon should judge (a) patency, (b) nonpatency, or (c) unclearness according to the conventional methods: (1) visual confirmation of the movement of lymph or blood at the anastomosis site; and (2) examination of patency—a method by which 2 tweezers are used to transiently generate a fluid-free condition between the tweezers by stemming the venous flow with them, followed by the confirmation of fluid movement at the anastomosis site after lifting only the tweezer that was placed on the peripheral side. After confirmation according to the conventional methods, the surgeon should subcutaneously inject 0.2 mL of ICG 2.5 mg/mL solution into the site distal from each anastomosis site and should judge (a) patency, (b) nonpatency, or (c) unclearness based on the findings of ICG fluorescent lymphography. The schedule for the study visits and data collection is summarized in Table 1.

**Table 1:** Schedules for the conduct of the clinical trial. The schedules to conduct monitoring, testing, and assessment are shown in the table below. The investigator or subinvestigator should conduct monitoring, testing, and others in accordance with the schedule.

	Consent acquisition	Screening phase		Investigational drug administration phase [assessment phase]		Investigational drug administration phase [treatment phase]		At the time of comple-tion Day 7	At the time of discon-tinuation
		Screening test		ICG (investigational drug)	Scintigraphy	Testing and treatment phase (hospitalization and control)			
	Visit Day 1 Weeks −26 to −9			Visit Day 2 Weeks −17 to −9	Visit Day 3 Weeks −17 to −1^a^	Day of imaging before surgery Day −1	Day of surgery Day 1		
Allowable range	–			–	*	+1	–	±1	+3
Written informed consent	●								
Primary registration			●						
Secondary registration					●				
History taking of the subject's background			●						
History taking of the primary disease, anamnesis, and complications			●						
Measurement of vital signs			●	●	●	●	●	●	●
Blood sampling (hematology, blood chemistry)^b^			●					●	●
Physical examination (measurement of the circumference of the upper or lower extremities of both sides)			●					●	●
Urinalysis			●						
Pregnancy test^c^			●						
Diagnostic ICG fluorescent lymphangiography				●					
^99m^Tc lymphoscintigraphy (the extremities of both sides)					●				
Photography of appearance			●						
Classification of International Society of Lymphology stages			●						
ICG fluorescent lymphangiography before surgery						●			
ICG fluorescent lymphangiography during surgery (examination of patency)							●		
Monitoring of adverse events^d^				●					●
Check of defects				●					●
Check of concurrent drugs and concurrent therapies			●						●

a Visit Day 3 (day of scintigraphy) should be the same day as visit Day 2 (day of ICG lymphangiography) or within 8 weeks.

b Hematology: WBC, RBC, Hb, Hct, Plt, differential leukocyte counts (neutrophils, lymphocytes, monocytes, basophils, and eosinophils), and prothrombin time Blood chemistry: AST, ALT, ALP, γ-GTP, LDH, T-Bil, D-Bil, TP, Alb, Glu, T-Cho, HDL, TG, UA, BUN, Cre, Na, K, Cl, ChE, and HbA1c.

c Not required for patients about whom the potential of pregnancy can be denied (e.g., women who underwent hysterectomy and menopausal women).

d All of adverse events, which occurred from the first subcutaneous injection of ICG in the investigational drug administration phase [assessment phase] to the completion of the trial period (Day 7).

## 3 Outcomes

### 3.1 Primary endpoint

The primary endpoint of this study is to calculate the identification rate of the lymphatic vessels at the incision site only in patients who are indicated for LVA based on the following requisites: the radiographic evidence of lymphatic stasis; and the presence of the anastomosable lymphatic vessels. Therefore, the investigational drug should be administered in the primary and secondary registration. Patients who meet the eligibility criteria for the primary registration will be assessed in the assessment phase and will undergo ICG fluorescent lymphography and lymphoscintigraphy. LVA will not be conducted for those whom the procedure is not indicated, and the study will be discontinued. Furthermore, patients who meet the eligibility criteria for the secondary registration will be assessed in the treatment phase and will undergo preoperative and intraoperative ICG fluorescent lymphography. The third-party expert panel independent from the present clinical trial should make the final judgment based on the triad of fluorescent lymphograms before surgery, light images before surgery, and the photographs of the lymphatic vessels that were taken after skin incision during surgery.

### 3.2 Efficacy secondary endpoints

(1) Sensitivity and specificity of ICG lymphography for specifying patients to whom LVA is indicated when using ^99m^Tc lymphoscintigraphy as one of the standard diagnostic methods

The specification of patients with lymphoedema for LVA should be judged based on the findings of ^99m^Tc lymphoscintigraphy and ICG fluorescent lymphography at the time of assessing the disorder, and the sensitivity and specificity of ICG lymphography when using ^99m^Tc lymphoscintigraphy as one of the standard diagnostic methods should be calculated from the results of the examinations. In this study, the anatomy of the lymphatic vessels on the contralateral side of the affected arm of unilateral breast cancer patients should also be assessed according to the same assessment criteria as those for the affected side because breast cancer patients with unilateral lymphoedema often present the lymphatic dysfunction of the contralateral “unaffected” arm [28].

The criteria for the indication of LVA based on ^99m^Tc lymphoscintigraphic findings: the case in which the lymphatic vessels of the auxiliary lymph nodes in the upper extremities and of the inguinal lymph nodes in the lower extremities are not depicted or depicted just unclearly, as well as dermal back flow was depicted in the extremities (categorized to types II, III, and IV according to the classification of Maegawa et al.); the case in which the movement of the tracer from the injection site cannot be found (categorized to type V according to the classification of Maegawa et al.); and the case in which another atypical dermal back flow is depicted (corresponding to unclassifiable according to the classification of Maegawa et al.) [12,13] Lymphedema in the status of surgical indication should be recorded as “negative” in other cases.

The criteria for the indication of LVA based on ^99m^Tc ICG fluorescent lymphographic findings: the case in which obvious dermal back flow is found in the extremities, and the case in which any movement of ICG from the injection site cannot be found. In this study, the splash pattern of lymphoedema—which has not necessarily been reported as a sign of irreversible chronic lymphoedema—should not considered as an indication for LVA.

LVA should not be indicated when the target extremity consists of only the region of the linear pattern and the area where a fluorescent image is not found.

The above should be based to calculate the sensitivity and specificity of the ability of ICG fluorescent lymphography to determine lymphoedema for LVA when using lymphoscintigraphy as one of the standard diagnostic methods.

The third parties, who are specialists in the surgical treatment of lymphoedema outside the study site, should judge centrally that the images of ^99m^Tc lymphoscintigraphy and ICG fluorescent lymphography are positive or negative for patients to whom LVA is indicated, respectively.

(2) The outer diameter of the identified lymphatic vessels (the number of the overlapped lymphatic vessels and the outer diameter of the respective vessels), as well as the distance from the skin surface to the lymphatic vessels (during surgery)
(3) Usefulness of ICG fluorescent lymphography when examining the presence or absence of the patency of the anastomosis site during LVA (during surgery)

By using the conventional methods and ICG fluorescent lymphography, the surgeon should judge whether or not the latter successfully determined the presence or absence of LVA site patency according to the following 4-level criterion:

(a) very useful: the case in which the judgment according to the conventional methods does not coincide with the judgment by ICG lymphography, with the determination that the judgment by ICG lymphography is correct;
(b) useful: the case in which the judgment by ICG lymphography coincided with the judgment according to the conventional methods when the judgment according to the conventional method indicated patency or nonpatency;
(c) not much useful: the case in which the judgment by ICG lymphography coincided with the judgment according to the conventional methods when the judgment according to the conventional methods indicated unclearness; and
(d) not useful at all: the case in which the judgment according to the conventional methods does not coincide with the judgment by ICG lymphography, with the determination that the judgment according to the conventional methods is correct.

### 3.3 Safety secondary endpoints

Presence or absence of adverse events and defects.

### 3.4 Data management, monitoring, safety and auditing

In collecting the data, the investigator should prepare the case report form by using software that meet the requirements in 21 CFR Part 11, Ministerial Ordinance GCP, and ER/ES guideline.

In the quality control of the clinical trial, the monitor should conduct the source document verification as appropriate and should confirm that the present clinical trial is conducted in compliance with the procedures on the medical institution's operations related to the present clinical trial, the latest protocol, and GCP.

To assure that the clinical trial was conducted appropriately, as well as the preparation, recording, and reporting of data were made appropriately in compliance with the protocol and GCP, an auditor who is independent from the departments related to the clinical trial including the department in charge of monitoring should conduct an audit at the medical institution and other facilities related to the conduct of the clinical trial and should confirm that quality control is conducted appropriately.

## 4 Statistical methods

The primary analysis set for the present clinical trial will be the full analysis set (FAS). Out of patients who should be included during the secondary registration, those falling under any of the following exclusion criteria will be excluded from the analysis: patients who severely violate the protocol; and patients whose images are of poor quality and cannot be interpreted (cases of test failure).

(1) Primary endpoint: the identification rate of the lymphatic vessels at the incision site based on ICG fluorescent lymphography (positive predictive rate). To date, no previous clinical study has mentioned the identification rate of the lymphatic vessels in the subcutaneous tissue. A multicentre collaborative confirmatory clinical trial of the sentinel lymph node biopsy for breast cancer was designed to consider a lymph vessel identification rate of 90% as being effective [26]. This was based on the fact that prior studies had reported a lymph node identification rate of 93%. The identification rate in the lymph node biopsy of breast cancer was 99.6% with respect to the investigational drug alone. The deviation of the lymph flow from normal anatomy is anticipated in the present clinical trial. However, a positive predictive rate of 90% should be established as the primary endpoint because an identification rate of 90% is required for demonstrating the usefulness of the study. In this study, the positivity rate and the Clopper-Pearson 95% confidence interval should be calculated. It should be confirmed that the positive rate is at least 90% and the lower confidence limit is at least 80%.
(2) Secondary endpoint-1: usefulness of ICG fluorescent lymphography in determining the indication for LVA to examine secondary lymphoedema. The usefulness of ICG fluorescent lymphography should be assessed as compared with ^99m^Tc lymphoscintigraphy—one of the standard diagnostic methods. Factors such as sensitivity, specificity, positive predictive rate, negative predictive rate, positive likelihood ratio, negative likelihood ratio, and accuracy should be calculated with an assumption that both the sensitivity and specificity of the lymphoscintigraphy-based judgement of lymphoedema are 1.0. Analyses should be made by using the standard 2 × 2 contingency table (lymphoedema positive, lymphoedema negative). The accuracy of the judgement of indication for LVA by ICG fluorescent lymphography should be defined as (the number of true positive patients + the number of true negative patients)/(the number of false positive patients + the number of false negative patients).
(3) Secondary endpoint-2: usefulness of ICG fluorescent lymphography in confirming the patency of the anastomosis site after LVA. The proportion of patients in whom ICG fluorescent lymphography was useful for identifying the presence or absence of patency after LVA.
(4) Presence or absence of adverse events and defects. The incidences and 95% confidence intervals of all adverse events and defects in the safety analysis population should be calculated. The Clopper–Pearson method should be used to calculate confidence intervals. Serious adverse events should be tabulated according to patients, and the incidences should be calculated according to system organ classes and preferred terms.

## 5 Ethics and dissemination

### 5.1 Research ethics approval and protocol amendments

Prior to the conduct of the present clinical trial, the trial will be approved by the institutional review board (IRB) at each of the participating institutions and will be conducted in accordance with GCP principles and the Declaration of Helsinki. The trial was notified and registered at PMDA and at the JMACCT registry (RCT2031190064).

### 5.2 Patient and public involvement

Patients of the public will be involved in the design and conduct of this trial. Once the trial has been published, participants will be informed of the results in a study newsletter suitable for a nonspecialist audience.

### 5.3 Informed consent

All participants will receive adequate information on the nature, purpose, possible risks and benefits of the trial, and alternative therapeutic choices, using the informed consent form approved by the IRB. The participants will be given ample time and opportunity to ask questions and to consider participation in the trial.

### 5.4 Confidentiality

To assure confidentiality, trial participants will be allocated a unique trial identification number throughout the trial.

## 6 Discussion

The usefulness of ICG fluorescent lymphography is well recognized among lymphatic microsurgeons because the imaging procedure was epoch-making for visualizing the lymphatic vessels that had been unobservable on the body surface without using a highly invasive method [[8], [9], [10], [11], [12], [13], [14], [15], [16], [17], [18], [19], [20], [21], [22], [23], [24], [25]]. Therefore, the need to objectively demonstrate the usefulness of ICG fluorescent lymphography in a prospective study has not been discussed. In recent years, however, new techniques (e.g., magnetic resonance imaging, ultrahigh-frequency ultrasound, and photoacoustic lymphography) have been reported. Hence, further improvements in the lymphatic vessel-depicting ability of these imaging modalities will be required in the future [[29], [30], [31]]. Whether ICG fluorescent lymphography, which is already in widespread use among lymphatic microsurgeons, is useful in identifying the lymphatic vessels suitable for LVA should be investigated in a prospective study before further research examines the usefulness of other new modalities in this regard. This clinical study is intended not to determine the depiction limits of ICG fluorescent lymphography but to record the thickness and depth of the lymphatic vessels for reference. Lymphatic flow on ICG fluorescent lymphograms is difficult to quantify. Although the doses and dosing concentrations of ICG have not been consistently reported in previous clinical studies, the results of a questionnaire-based survey—conducted through the Japan Society of Plastic Surgeons—at major medical institutions in Japan that are engaged in LVA were used for reference. Any adequate studies on the lymph vessel identification of the extremities were not available when determining the sample size of the present study. Therefore, the lymph vessel identification rate in a study on the sentinel lymph node biopsy of breast cancer [26] was used for reference. The PMDA admitted the above method to determine the sample size of this phase III clinical trial.

This trial does not consider the quantitative nature of the lymph flow velocity measurement [32]. Therefore, we standardized neither the location of the injection sites, nor monitoring time between ICG fluorescent lymphography and ^99m^Tc lymphoscintigraphy. The most appropriate protocol for ^99m^Tc lymphoscintigraphy in the literature has yet to be validated. The protocol of the present study was prepared based on the article published by one of the coauthors [7]; the article had 92 citations as of October 3, 2019, and forms the cornerstone for many clinical studies. In the present study, the number of injection sites is limited to 2 and scintigraphy is recommended to conduct at 120 min after tracer administration based on the review outcomes of patients with advanced lymphoedema.

ICG fluorescent lymphography, which allows the real-time monitoring of the lymphatic vessels in the examination room, is highly beneficial for both physicians and patients. Hence, the fact that ICG fluorescent lymphography causes less burdens and is less time-consuming to depict the lymphatic vessels than ^99m^Tc lymphoscintigraphy is of clinical relevance. Therefore, we did not use a fixed measurement time protocol for ICG fluorescent lymphography. Five injection sites for ICG fluorescent lymphography are presumably most efficient for identifying the lymphatic vessels based on a co-authors’ study in which lymphangiosomes in cadavers were scrutinized by ICG fluorescent lymphography [27]. These 5 sites are supposedly necessary to find, at the highest probability rate, the linear pattern of lymphoedema on ICG fluorescent lymphograms that can be used in LVA. Since ^99m^Tc lymphoscintigraphy is not intended to identify the individual lymphatic vessels, it is not useful to inject a radioisotope into these 5 sites in order to increase radiation exposure.

This study has several limitations. First, this study is intended to examine patients who are indicated for the surgical treatment of secondary lymphoedema and does not aim to examine patients about whom the cause of lymphoedema was determined (e.g., patients with venous disease or suspected primary lymphoedema). Since the primary endpoint of this study is to calculate the lymphatic vessel identification rate in LVA, we will enroll patients who are scheduled to undergo LVA based on their clinical findings. Patients, in whom venous disease is the primary cause of lymphoedema, should be excluded from this study because LVA is not indicated for them. In addition, the usefulness of LVA for patients with primary lymphoedema has not been proved. We will intend to examine the usefulness of ICG fluorescent lymphography in differentiating lymphoedema caused by lymphatic and venous diseases once the results from this study are obtained. Second, the present study is designed neither to provide the long-term outcomes of LVA, nor to verify the safety of LVA. Any conclusive and globally acceptable views are still not obtained with respect to its superiority to therapeutic modalities including conservative therapy for lymphoedema and to its applicability, constituting a great clinical challenge to be addressed [[33], [34], [35]]. The present clinical study is intended to preliminarily verify the usefulness of ICG fluorescent lymphography in assessing secondary lymphoedema and to determine the accuracy of depicting the collecting lymphatics and dermal back flow—an indication for LVA. We expect the present study be a foundation for future clinical studies to assess the surgical treatment of secondary lymphoedema. Third, patients with subclinical lymphoedema are excluded from the present study although the prophylactic treatment of and surgical intervention for them have been reported [15,36]. Since the severity of lymphoedema—based on which LVA is indicated for patients with subclinical lymphoedema—remains to be determined, which causes a dispute. Therefore, this study aims to examine patients with lymphoedema who show dermal back flow—a common indication for surgery. Fourth, 2 sessions of ICG fluorescent lymphography, one each in the assessment and treatments phases, and should be separated by an interval of at least 8 weeks to avoid the effects of residual ICG. On the other hand, the interval between ICG fluorescent lymphography and ^99m^Tc lymphoscintigraphy in the assessment phase should be shorted to the extent possible. We consider that the ≤ 8-week-interval of ICG fluorescent lymphography and ^99m^Tc lymphography in the assessment phase is acceptable because the present study enrolls patients with chronic lymphoedema of the extremities who are undergoing conservative therapy. In the case that significant changes (e.g., the disappearance of the linear pattern of lymphoedema on ICG fluorescent lymphograms in the treatment phase) occurred, we will analyze the relevant changes in a short interval.

## Data availability statement

Not applicable.

## Contributors

NU designed the original concept. The protocol was written by SA and NU, and was critically reviewed by all authors. SA drafted the manuscript, and all authors contributed constructive comments on the manuscript and approved the final paper. SA and NM are the guarantors for the publication and are fully responsible for the paper protocol.

## Funding

The present clinical trial plans to be conducted under funding from 10.13039/501100010957Hamamatsu Photonics K.K. This work was supported in part by the project “Assessment of the utility of indocyanine green fluorescent lymphography in diagnosing lymphoedema” from 10.13039/100009619Japan Agency for Medical Research and Development; JMA-CCT-A-2704 (awarded to NU).

## Patient consent

Obtained.

## Data sharing statement

The authors confirm that the data supporting this study will be provided as final results and as supplementary material accompanying the manuscript of the study's final results. Raw data will be generated at Chiba University Hospital. Derived data supporting the findings of this study will be available from the corresponding author (SA) on request.

## Declaration of competing interest

The present clinical trial plans to be conducted under funding from Hamamatsu Photonics K.K. The possibility that conflict of interest occurs with respect to the conduct of the clinical trial and its outcomes should be controlled appropriately based on the conflict of interest management policies of each medical institution.
